# Birooted Mandibular Canine: A Systematic Review and Meta-Analysis

**DOI:** 10.3390/jcm15093381

**Published:** 2026-04-28

**Authors:** Amelia Hoppe, Kamila Chęcińska, Maciej Chęciński, Natalia Turosz, Maciej Sikora

**Affiliations:** 1 Department of Oral Surgery, Preventive Medicine Center, Komorowskiego 12, 30-106 Cracow, Poland; amelia.a.hoppe@gmail.com; 2 National Medical Institute of the Ministry of the Interior and Administration, Wołoska 137 Str., 02-507 Warsaw, Poland; maciej.checinski@pimmswia.gov.pl (M.C.); natalia.turosz@pimmswia.gov.pl (N.T.); maciej.sikora@pimmswia.gov.pl (M.S.); 3 Department of Maxillofacial Surgery, Hospital of the Ministry of the Interior and Administration, Wojska Polskiego 51, 25-375 Kielce, Poland; 4 Department of Biochemistry and Medical Chemistry, Pomeranian Medical University, Powstańców Wielkopolskich 72, 70-111 Szczecin, Poland

**Keywords:** mandibular canine, tooth morphology, root anatomy, anatomical variation

## Abstract

**Background/Objectives**: Birooted mandibular canines are a rare but clinically significant variation that is often underdiagnosed and may complicate dental treatment. The aim of this review was to conduct a comprehensive meta-analysis to estimate the prevalence of birooted and multirooted mandibular canines, and to address the research question of whether geographical region, sex, or side predilections significantly influence these anatomical variations. **Methods**: A systematic search was performed on 4 September 2025 across five databases: BASE, Scopus, PubMed, DOAJ, and Scielo. Studies on permanent mandibular canines with at least 10 cases, assessed radiologically or in vitro, were included. Reports without detailed root number data or based only on two-dimensional imaging were excluded. Meta-analysis estimated prevalence and odds ratios by sex and geographic region, with results shown in tables and graphs. Risk of bias was assessed using the Joanna Briggs Institute appraisal tools. **Results**: Eighteen studies met the inclusion criteria, of which seventeen were classified as population studies and one case as a series. The pooled prevalence of birooted mandibular canines was 2.71%. A female predilection was statistically significant in four modern cohorts. Only one case of a three-rooted mandibular canine was documented. No clinical outcome data were available regarding endodontic success or extraction complications. **Conclusions**: Birooted mandibular canines represent a relatively common anatomical variant, especially in females and on the right side of the jaw. However, clinical implications related to treatment outcomes remain underreported, emphasizing the need for future clinical and radiological investigations.

## 1. Introduction

Deviations from typical dental anatomy are not only an intriguing radiological issue but also a real challenge in direct patient care. Anatomical variations make all types of dental therapeutic procedures that assume tooth preservation difficult, including endodontic treatment, orthodontic procedures, periodontal procedures, and prosthetic replacements [1,2].

Initial difficulties can arise during the evaluation of radiographic images, as anatomical irregularities may not be clearly visible on two-dimensional imaging [3,4]. This should be taken into consideration, as an accurate assessment of the number and morphology of roots is crucial for successful treatment [3,4]. A Cone Beam Computed Tomography (CBCT) scan might be beneficial in case a standard orthopantomogram does not provide enough detail [5,6]. With the development of modern radiological equipment and growing knowledge in the field, exposure doses during diagnostic examinations are decreasing without compromising the quality of the obtained clinical evaluation [7,8]. Consequently, many patients present with comprehensive dental radiological documentation, often including CBCT images. Existing imaging should be assessed first, with any additional radiological diagnostic procedures planned, ensuring the lowest possible radiation dose while still achieving diagnostically interpretable results.

Deformations and bifurcations of tooth roots and the presence of additional roots are of particular importance in endodontics [9,10]. Additional roots may contain narrow, curved, complex root canals, making instrumentation and irrigation difficult or even impossible [10,11]. Further difficulties may arise at the stage of root canal obturation [10,12,13]. Improper preparation, combined with unsound filling, consequently leads to the failure of root canal treatment [14,15,16].

Not only preservation but also extraction of atypically rooted teeth requires careful planning. Extraction of a tooth with atypical root formation can be more challenging even for an experienced surgeon [17,18,19]. Root position and curvature can increase the difficulty of extraction and prolong the procedure, often requiring a higher dose of anesthesia [20,21,22,23]. Additionally, extending the duration of the intervention significantly reduces patients’ comfort.

Diagnostics based only on two-dimensional imaging may result in unexpected complications and require widening of surgical access. Nonetheless, despite extensive preparation of both soft and hard tissues, atypical root anatomy may provoke an unexpected extraction path leading to intra-alveolar root fragmentation, further complicating the procedure. Moreover, complicated extractions lead to significant bone loss [24]. In turn, the lack of bone tissue exposes the patient to additional augmentation procedures in preparation for implant placement [25,26,27].

The mandibular canine, typically the longest tooth in the lower dental arch, predominantly presents with a single root. Root bifurcation or an additional root in the case of lower canines is particularly uncommon. However, due to the importance of this tooth for lateral guidance, stabilization of fixed prosthetic restorations and removable dentures, the loss of a lower canine due to the failure of conservative treatment can be exceptionally troublesome [28,29,30]. Lower canines are usually the last teeth that make up the residual dentition of the lower arch and are crucial for the stabilization of a removable denture. Their loss, often resulting in edentulism of the lower arch, does not bode well for maintaining a lower denture [31,32,33]. Suppose this problem is aggravated by the lack of bone tissue for implantation in the medial part of the alveolar process of the mandible in relation to the mental foramen. In that case, the patient may be sentenced to use an unstable lower complete denture or transposition of the inferior alveolar nerve in the course of implantoprosthetic rehabilitation [31,34,35].

The aim of this study was to systematically review the available literature and conduct a meta-analysis to estimate the prevalence of additional roots in mandibular canines, as well as to assess potential differences according to sex, geographical region, and side of the jaw. The null hypothesis assumed the absence of significant differences in prevalence across sex, geographical regions, and sides of the jaw.

## 2. Materials and Methods

This systematic review with meta-analysis was pre-registered in PROSPERO (ID: CRD420251138164) and conducted according to PRISMA 2020 [36,37]. 

### 2.1. Eligibility Criteria

Studies were included in the review if they met the following criteria. The subject of the study was required to be two-rooted or multi-rooted anatomical variations in the mandibular canines. In particular, reports on accessory or bifurcated roots were sought. Papers on deciduous teeth were excluded due to the developmental differences in the primary dentition.

Human subject studies with assessment of the root number of permanent mandibular canines were included. Both observational and experimental studies were eligible, including studies reporting a series of at least 10 cases. Therefore, descriptions of isolated occurrences of anomalies (case reports and small case series) were excluded. Root number could be assessed using any method considered appropriate by the authors, including direct evaluation of extracted teeth or radiological imaging. However, studies lacking sufficient details regarding this parameter were excluded. Nevertheless, studies based solely on two-dimensional imagining were excluded due to the risk of inaccurate root assessment. The review included articles published in the last 40 years (from 1985 to 2025) in English or other languages with an available English translation by the authors, the editors or by the Google Translate tool (Google LLC, Mountain View, CA, USA) were accepted. Preprints, conference proceedings, reviews, and papers not published in journals with a specific International Standard Serial Number (ISSN) were rejected.

Studies were grouped for synthesis according to the specific outcome measures reported. All studies meeting the inclusion criteria were summarized in a table with reported results. Studies classified as case series were excluded from the quantitative synthesis and were not included in the pooled population. Furthermore, studies providing data on the prevalence of birooted canines stratified by sex or by side of the jaw were analyzed in subgroups.

### 2.2. Information Sources and Search Strategy

Searches were conducted using the following engines: (1) Bielefeld Academic Search Engine (BASE), (2) Scopus, (3) the National Library of Medicine PubMed, (4) Directory of Open Access Journals (DOAJ), and (5) Scientific Electronic Library Online (SciELO).

The engine query was developed based on the eligibility criteria. After the process of developing, supplementing and refining the query, it finally adopted the following content: (“additional root” OR “accessory root” OR “bifurcated root” OR birooted OR “double-rooted” OR “extra-rooted” OR “multi-rooted” OR “root bifurcation” OR “root duplication” OR “supernumerary root” OR “supernumerary roots” OR “two-rooted”) AND (inferior OR lower OR mandibular) AND (canine OR cuspid). The final searches were conducted on 4 September 2025.

### 2.3. Selection Process, Data Collection Process, and Data Items

Research selection and removal of duplicates were performed using the Rayyan platform [38]. Identified records were manually screened by two authors independently, based on their titles and abstracts, against the eligibility criteria. Cohen’s kappa was calculated to measure the inter-reviewer agreement. Those articles that met the criteria underwent a manual, non-automated full-text assessment. All articles for which a discrepancy occurred were included for full-text review.

The following information characterizing individual studies and outcome data was obtained from the content of the reports: (1) first author and year of publication, (2) type of study qualification (population or cases of birooted lower canines), (3) sample size (for population studies), (4) numbers of males and females within the sample, (5) number of birooted lower canines identified in the sample (distinguishing values for particular sexes and sides of the jaw), (6) numbers right and left lower canines within the sample, and (7) number of three- and more-rooted canines identified in the sample.

### 2.4. Study Risk of Bias Assessment, Effect Measures, and Synthesis Methods

The risk of bias was assessed using critical appraisal tools proposed by the Joanna Briggs Institute, dedicated to particular types of research, independently by two authors [39]. Any discrepancies between the reviewers were resolved through discussion until consensus was reached.

The collected results were presented in tabular form using the Google Workspace package (2025 version, Google LLC, Mountain View, CA, USA). The odds ratio values, with 95% confidence intervals (95% CI), were calculated in the R environment (version 4.5.1, R Core Team, R Foundation for Statistical Computing, Vienna, Austria) using the epitools package (version 0.5.10.1) [40] and Wald method, plotted in forest plots using the Python programming language (Python Software Foundation, Wilmington, DE, USA, version 3.14) with the matplotlib (Matplotlib Development Team, University of California, Irvine, CA, USA, version 3.10.7), and seaborn (Michael Waskom, New York University, New York, NY, USA, version 0.13.2) libraries.

The statistical significance of the odds ratio was determined using Fisher’s exact test at a significance level of α = 0.05.

### 2.5. Certainty Assessment

The GRADE system was applied to assess the quality of collected evidence.

## 3. Results

**Figure 1:**
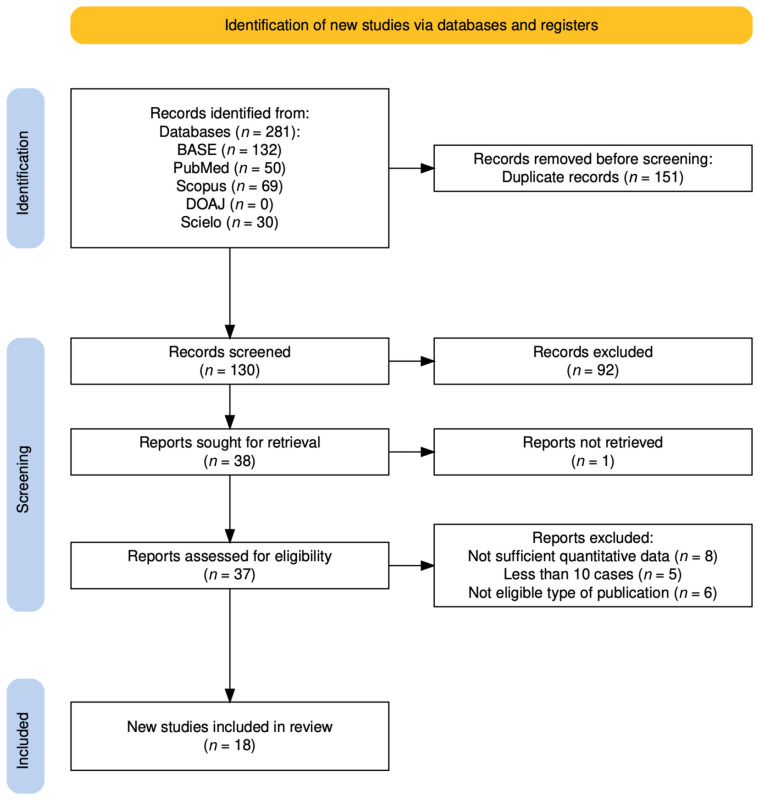
Flow diagram. Adapted from the PRISMA 2020 flow diagram (© The PRISMA Executive), distributed under the Creative Commons Attribution 4.0 (CC BY 4.0) license.

Searches in medical databases yielded 281 records. Of those, 151 duplicates were detected. Throughout manual screening of 130 remaining articles, 38 whose titles and abstracts suggested potential compliance with the eligibility criteria were qualified for full-text evaluation. The Cohen’s kappa coefficient for inter-reviewer agreement during manual screening was 0.82, which is commonly interpreted as indicating near perfect agreement (values 0.81–1.00), with concordant decisions in 93% of cases. Unfortunately, despite the effort made, access to the full text of one record could not be retrieved. The remaining articles underwent full-text assessment, resulting in further exclusions (Table A1). Finally, 18 studies were included in the synthesis (Table 1). The subsequent stages of the selection process are illustrated in the flow diagram (Figure 1) [41]. All records excluded at the stage of full-text assessment were listed in a Table A1, together with the reasons for exclusion.

The systematic review included 17 studies classified for analysis purposes as population-based studies and 1 case series. No data were obtained on the effectiveness of endodontic treatment of two- and more-rooted lower canines. No information was also collected on the course of extraction of teeth presenting the discussed anatomical variance. Therefore, the presentation of columns on this topic in the table was omitted (Table 1).

**Figure 2:**
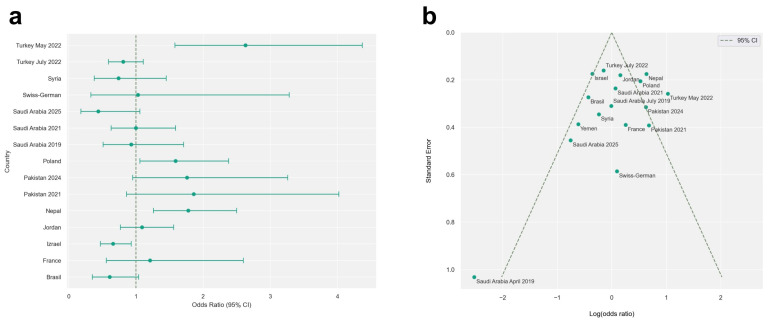
Analysis by geographical region. (**a**) Odds ratio—forest plot. Values to the right of the vertical line (>1) indicate higher prevalence of birooted mandibular canines in specific populations. Horizontal lines represent 95% confidence intervals (95% CI). (**b**) Funnel plot assessing publication bias. The vertical axis represents the standard error (SE), while the horizontal axis represents the log odds ratio. Values less than 0 indicate lower prevalence of birooted canines in a specific study compared to the overall pooled population, while values above 0 indicate higher prevalence.

The results of the meta-analysis are illustrated using forest plots and funnel plots (Figure 2). Among the 11,006 mandibular canines examined, 299 had additional roots, with a prevalence of birooted canines of 2.71%.

**Figure 3:**
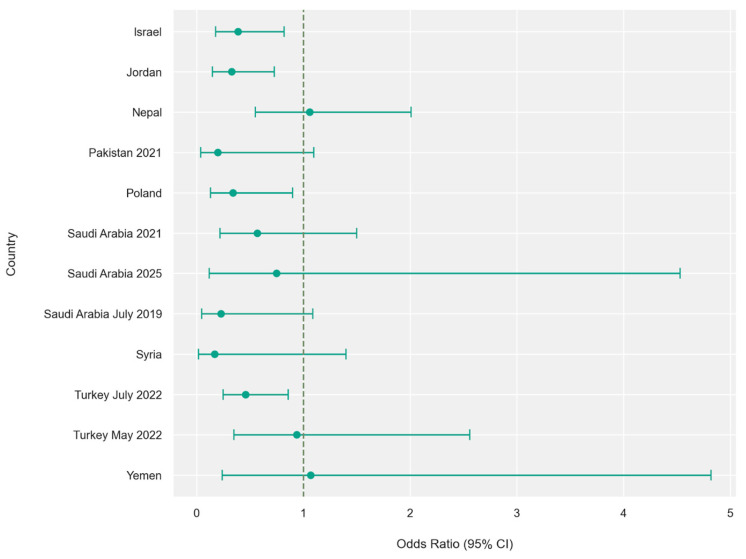
Odds ratio by sex (male/female ratio) per country—forest plot. Values to the right of the vertical line (>1) indicate higher prevalence of birooted mandibular canines in males, while values to the left (<1) indicate higher prevalence in females. Horizontal lines represent 95% confidence intervals (95% CI).

**Figure 4:**
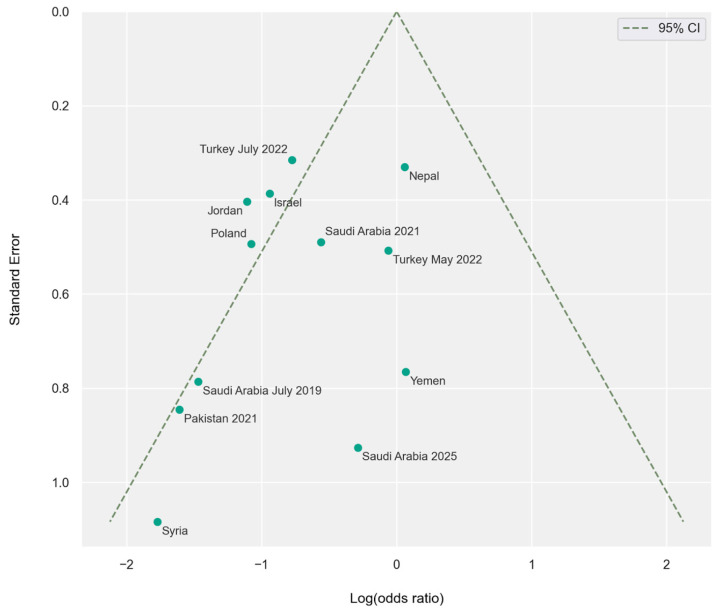
Funnel plot illustrating odds ratio regarding sex, per country. The vertical axis represents the standard error (SE), while the horizontal axis represents the log odds ratio. Values less than 0 indicate higher prevalence of birooted canines in females compared to males, while values above 0 indicate higher prevalence in males.

Sex-stratified analysis revealed a statistically significant difference between males and females (*p* = 0.03). An amount of 180 of 262 birooted canines occurred in females with a pooled male-to-female odds ratio of 0.50 (95% CI: 0.39–0.65; *p* < 0.001) (Figure 3 and Figure 4).

**Figure 5:**
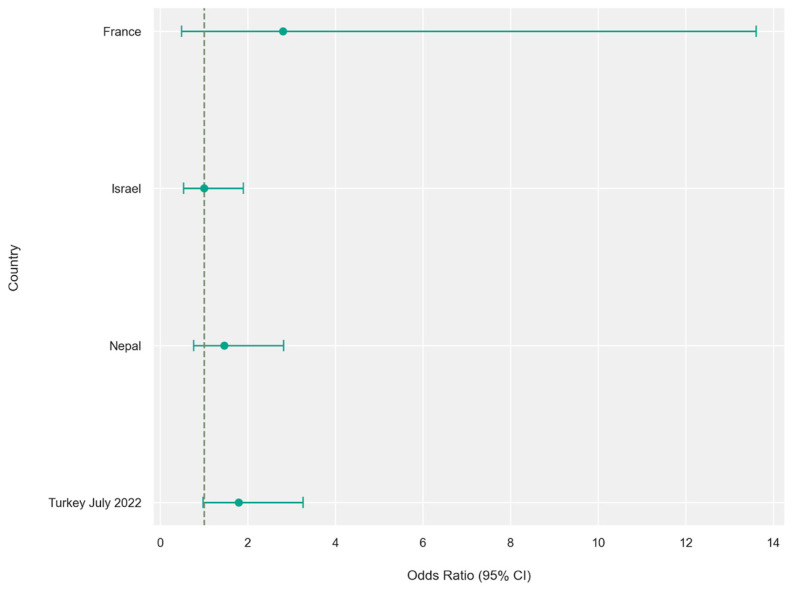
Odds ratio by side (per country)—forest plot. Values to the right of the vertical line (>1) indicate higher prevalence of birooted canines in the right side of the mandible, while values to the left (<1) indicate higher prevalence in the left side. Horizontal lines represent 95% confidence intervals (95% CI).

**Figure 6:**
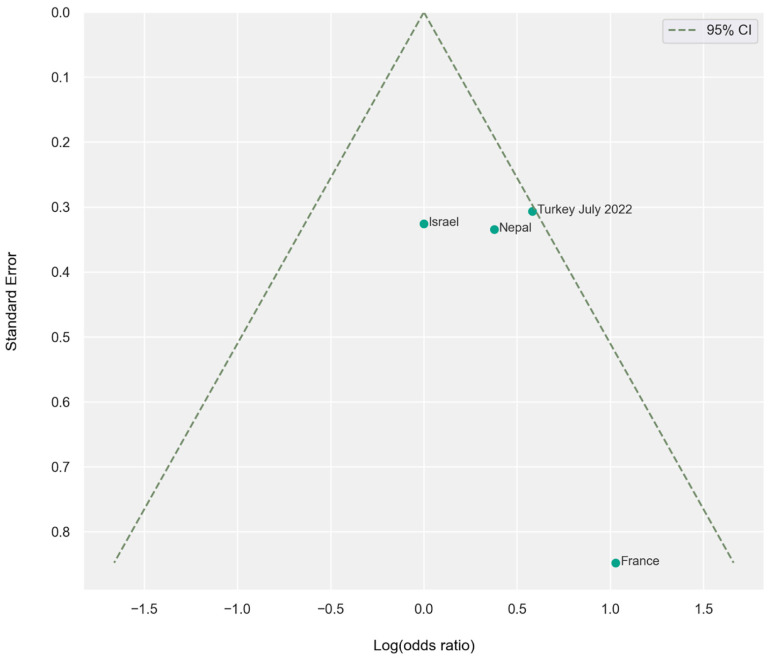
Funnel plot illustrating the odds ratio by side per country. The vertical axis represents the standard error (SE), while the horizontal axis represents the log odds ratio. All studies on the right side with values higher than 0 reported a higher prevalence of birooted canines on the right side of the mandible, while the study conducted in Israel, with a value equal to 0, indicates an equal prevalence of birooted canines on the left and right sides.

Laterality analysis of 4963 canines revealed a modest right-sided predominance (right/left = 77:54; OR = 1.44, 95% CI: 1.01–2.05; *p* = 0.051), although this trend was not consistently observed across individual studies (Figure 5 and Figure 6).

The risk of bias assessment, as summarized in Table A2, Table A3, Table A4, Table A5 and Table A6, did not reveal major methodological concerns, and no studies were excluded on this basis. However, the GRADE assessment indicated that the certainty of evidence regarding all assessed outcomes was rated as very low due to serious inconsistency observed across all outcome groups. The GRADE summary of findings table is provided in Table A7.

## 4. Discussion

### 4.1. Interpretation

According to the data from the identified and included studies, the reported incidence of birooted lower canines in the contemporary population ranges from 0.22% to 7.23%, with a mean prevalence of 2.71%. These findings indicate that although birooted mandibular canines are a relatively rare anatomical variation, their prevalence is sufficient for them to be encountered in routine clinical practice. This outcome appears consistent with findings from Martins et al. (2024), which reported the global prevalence of birooted canines at 1.9%, a comparable result [60].

Among studies included in the synthesis, three reported a statistically significant higher incidence of birooted lower canines, with the highest prevalence reported by Okumuş et al. at 7.23% [42,52,53]. However, it should be noted that in the same year, another study conducted in Turkey, on a larger sample, did not demonstrate a similarly high frequency of this anomaly, despite comparable study conditions [43]. One study by Al-Dahman et al., in particular, showed a wide range of results and a high standard error, raising concerns regarding the reliability of its findings [58]. However, no obvious explanation for this finding was identified. The number of canines examined was not substantially lower, as the sample size corresponded to the median of the study groups included in the review, and methodological conditions were comparable to those of the other included investigations. Notably, after exclusion from the pooled population of the outstanding study by Al-Dahman et al., the prevalence of birooted mandibular canines ranges from 1.28% to 7.23% in contemporary populations, with a mean prevalence of 2.81%.

Furthermore, an archeological study by Lee et al. suggests that a higher incidence of birooted lower canines was typical in the historical Spanish population and may have been considerably higher in the past. Among 295 examined, 27 individuals exhibited birooted mandibular canines, and of the 19 assessed bilaterally, 31.6% was present on both sides [61]. This also aligns with the previously cited review, which reported that the Spanish population has a prevalence of birooted mandibular canines of 6.7% [60].

Most of the studies that take into account patient sex indicate that birooted lower canines are more common in women, which is consistent with the review conducted by other authors [60]. Statistical significance of female predilection is observed in four contemporary population studies [42,43,55,57]. Although two articles suggested a higher prevalence of birooted mandibular canines in males, neither of these results could be considered statistically significant [53,59]. The odds ratio in the pooled population was 0.50 (95% CI: 0.39–0.65); however, significant heterogeneity in results was observed. An attempt was made to analyze the study, excluding the outliers. Regarding the sexual dimorphism subanalysis, the seven most homogenic outcome studies were grouped together, and additional analysis was performed [42,43,44,52,53,55,57]. The odds ratio for this subset was 0.53 (95% CI: 0.40–0.71). One study was excluded from the subanalysis as it reported no cases of tworooted canines in women, preventing calculation of odds ratios, standard errors, and its inclusion in forest and funnel plots performed in this analysis for the remaining studies [58].

Only four studies reported information about the side of the jaw on which the birooted canine was located and the location of the other canines examined [43,50,53,57]. None of the included studies found this anatomical variant to be more frequent on the left side. Definitive asymmetry could not be confirmed in any individual studies, as none of the reported differences achieved statistical significance. However, the odds ratio of the pooled population was calculated at 1.44 (95% CI: 1.01–2.05), indicating a marginally significant predilection to the right side. Due to the relatively high standard error observed in one study, by Monsarrat et al., an additional analysis was performed, excluding these findings [50]. The odds ratio in the subgroup of three studies was 1.40 (95% CI: 0.97–2.00), suggesting that more homogeneous studies fail to demonstrate statistically significant asymmetries in the frequency of birooted mandibular canines. A possible explanation for this discrepancy could be the markedly smaller sample of examined canines in this study compared to the other three. Notably, three additional studies recorded the side of birooted canines but did not provide corresponding information for the other canines, preventing their inclusion into the subanalysis [42,49,54].

In the entire material, only one case of three-rooted lower canine teeth was identified. However, it was reported in a case series, no conclusions regarding the incidence of this phenomenon could be drawn [45].

Although statistically significant differences in prevalence across sex, geographical regions, and sides of the jaw were found, the null hypothesis formulated at the beginning of the research could not be definitively rejected, due to the very low certainty of the evidence, rated according to the GRADE system.

Identifying an atypical number of roots in mandibular canines may significantly impact treatment success. The assumption was that the previous approach to endodontic treatment and applied extraction techniques for those teeth would be documented in the included articles with appropriate evaluation. Unfortunately, we were unable to extract any data relevant to those aspects.

### 4.2. Limitations

Despite searching five medical databases using a high-sensitivity, low-specificity query, only a limited number of eligible studies were identified. Additionally, only four studies provided information on the laterality of assessed teeth, sufficient for the subanalysis, which limits the credibility of the outcomes. Furthermore, sample sizes were generally limited; only three included studies analyzed populations larger than 1000 canines, while another three assessed samples exceeding 500 canines. Given the low prevalence of this anatomical variant (2.71%), smaller samples may yield less precise estimates. There was heterogeneity in outcomes in both the overall meta-analysis and subgroup analysis. Simultaneously, due to the low certainty of the evidence, firm conclusions could not be made. It should be recognized that the topic of birooted lower canine is not significantly represented in the scientific literature. Furthermore, the included studies completely failed to address clinician-relevant complications of extraction and endodontic treatment.

### 4.3. Strengths

This study was conducted in accordance with PRISMA 2020 guidelines and was preregistered in PROSPERO. A comprehensive search with a refined query was conducted across multiple databases without language restriction, minimizing the risk of missing relevant data. The screening process was performed by two independent researchers and the inter-reviewer agreement was rated as near perfect, ensuring reproducibility of findings. Synthesizing available evidence provides a baseline for future large-scale epidemiological studies.

### 4.4. Future Perspectives

It seems reasonable to conduct further radiological studies, which will allow for increasing the total sample of the meta-analysis in the future. Further investigation of the anatomical characteristics of this anomaly should be considered due to the lack of comprehensive research available.

## 5. Conclusions

In conclusion, this comprehensive systematic review and meta-analysis, based on 18 modern population-based studies, demonstrated that additional roots in mandibular canines represent a relatively common anatomical variation, with an overall prevalence of 2.7% (approximately 1 in 37 mandibular canines). With respect to the prespecified subgroup analysis, the null hypothesis of no difference in prevalence could not be upheld. Sex-stratified analysis revealed a female predominance, with 180 of 262 birooted canines occurring in females. Analysis of laterality in 4963 canines demonstrated a marginally significant right-sided predominance. Regardless of overall findings, those conclusions were not consistently supported by individual studies and should therefore be interpreted with caution.

## Figures and Tables

**Table 1:** Source data and outcomes.

First Author, Publication Year, Identifier	Qualification Type (Population/Cases of Birooted Canines)	Number of Mandibular Canines (in M/F)	Birooted Canines	Odds Ratio per Population (95% CI, *p*)	Birooted Canines in M/F	M/F Odds Ratio (95% CI, *p*)	Number of R/L Canines	Birooted R/L Canines	R/L Odds Ratio (95% CI, *p*)	Three-Rooted (and More-Rooted) Canines
Piskórz, 2023, 10.17219/dmp/147758 [42]	Population (Poland)	600 M: 236 F: 364	27 (4.50%)	1.69 LCL: 1.13 UCL: 2.53 *p* = 0.01	M: 5 F: 22	0.34 LCL: 0.13 UCL: 0.90 *p* = 0.03	R: N/S L: N/S	R: 15 L: 12	N/A	0 (0)
Aydin, 2022, 10.1007/s11282-022-00637-8 [43]	Population (Turkey)	2000 M: 1000 F: 1000	47 (2.35%)	0.86 LCL: 0.63 UCL: 1.18 *p* = 0.41	M: 15 F: 32	0.46 LCL: 0.25 UCL: 0.86 *p* = 0.02	R: 1000 L: 1000	R: 30 L: 17	1.79 LCL: 0.98 UCL: 3.26 *p* = 0.08	0 (0)
Almohaimede, 2021, 10.1155/2021/5574512 [44]	Population (Saudi Arabia)	694 M: 295 * F: 399 *	20 (2.88%)	1.07 LCL: 0.67 UCL: 1.69 *p* = 0.72	M: 6 F: 14	0.57 LCL: 0.22 UCL: 1.50 *p* = 0.36	R: 347 L: 347	N/S	N/A	0 (0)
Beltes, 2019, 10.1111/aej.12305 [45]	Case series	78 M: N/S F: N/S	77 (N/A)	Excluded: case series	N/A	N/A	N/A	N/A	N/A	1 (0)
Versiani, 2011, 10.1111/j.1365-2591.2011.01879.x [46]	Population (Brasil †)	793 M: N/S F: N/S	14 (1.77%)	0.65 LCL: 0.38 UCL: 1.11 *p* = 0.13	N/S	N/A	N/S	N/S	N/A	0 (0)
Doumani, 2020, 10.4103/jfmpc.jfmpc_655_19 [47]	Population (Syria)	418 M: 172 F: 246	9 (2.15%)	0.79 LCL: 0.40 UCL: 1.55 *p* = 0.64	M: 1 F: 8	0.17 LCL: 0.02 UCL: 1.40 *p* = 0.09	N/S	N/S	N/A	0 (0)
Khan, 2021, 10.37723/jumdc.v12i1.483 [48]	Population (Pakistan)	134 M: 86 F: 48	7 (5.22%)	1.98 LCL: 0.92 UCL: 4.28 *p* = 0.10	M: 2 F: 5	0.20 LCL: 0.04 UCL: 1.10 *p* = 0.10	N/S	N/S	N/A	0 (0)
Mashyakhy, 2019, PMID: 31597794 [49]	Population (Saudi Arabia)	410 M: 197 F: 213	11 (2.68%)	0.99 LCL: 0.54 UCL: 1.82 *p* = 1.00	M: 2 F: 9	0.23 LCL: 0.05 UCL: 1.09 *p* = 0.06	N/S	R: 6 L: 5	N/A	0 (0)
Monsarrat, 2016, 10.1371/journal.pone.0165329 [50]	Population (France)	202 M: N/S F: N/S	7 (3.47%)	1.29 LCL: 0.60 UCL: 2.77 *p* = 0.51	N/S	N/A	R: 101 L: 101	R: 5 L: 2	2.58 LCL: 0.49 UCL: 13.61 *p* = 0.44	0 (0)
Mustafa, 2025, 10.1038/s41598-025-86277-4 [51]	Population (Saudi Arabia)	391 M: 260 F: 130 N/S: 1	5 (1.28%)	0.47 LCL: 0.19 UCL: 1.13 *p* = 0.11	M: 3 F: 2	0.75 LCL: 0.12 UCL: 4.53 *p* = 1.00	N/S	N/S	N/A	0 (0)
Okumuş, 2022, 10.15517/ijds.2022.51090 [52]	Population (Turkey)	235 M: 100 F: 135	17 (7.23%)	2.80 LCL: 1.69 UCL: 4.65 *p* = 0.00	M: 7 F: 10	0.94 LCL: 0.35 UCL: 2.56 *p* = 1.00	N/S	N/S	N/A	0 (0)
Shrestha, 2024, 10.33314/jnhrc.v21i3.4707 [53]	Population (Nepal)	780 M: 410 F: 370	39 (5.00%)	1.89 LCL: 1.34 UCL: 2.66 *p* = 0.00	M: 21 F: 18	1.06 LCL: 0.55 UCL: 2.01 *p* = 1.00	R: 390 L: 390	R: 23 L: 16	1.46 LCL: 0.76 UCL: 2.82 *p* = 0.32	0 (0)
Siddique, 2024, 10.12669/pjms.40.7.8744 [54]	Population (Pakistan)	222 M: N/S F: N/S	11 (4.95%)	1.87 LCL: 1.01 UCL: 3.47 *p* = 0.06	N/S	N/A	N/S	R: 6 L: 5	N/A	0 (0)
Taha, 2024, 10.1186/s12903-024-03934-2 [55]	Population (Jordan)	1114 M: 518 F: 596	35 (3.14%)	1.17 LCL: 0.82 UCL: 1.66 *p* = 0.37	M: 8 F: 27	0.33 LCL: 0.15 UCL: 0.73 *p* = 0.01	N/S	N/S	N/A	0 (0)
Wolf, 2021, 10.1038/s41598-021-00758-w [56]	Population (Swiss- German)	101 M: N/S F: N/S	3 (2.97%)	1.10 LCL: 0.35 UCL: 3.48 *p* = 0.76	N/S	N/A	N/S	N/S	N/A	0 (0)
Shemesh, 2016, PMID: 27295928 [57]	Population (Israel)	1981 M: 876 F: 1105	38 (1.92%)	0.70 LCL: 0.50 UCL: 0.99 *p* = 0.05	M: 9 F: 29	0.39 LCL: 0.18 UCL: 0.82 *p* = 0.01	R: 991 L: 990	R: 19 L: 19	1.00 LCL: 0.53 UCL: 1.90 *p* = 1.00	0 (0)
Al-Dahman, 2019, 10.4103/sej.sej_85_18 [58]	Population (Saudi Arabia)	454 M: 210 F: 244	1 (0.22%)	0.08 LCL: 0.01 UCL: 0.57 *p* = 0.00	M: 1 F: 0	N/A	N/S	N/S	N/A	0 (0)
Alhumaidi, 2025, 10.1007/s10266-024-00965-7 [59]	Population (Yemen)	477 M: 197 F: 280	7 (1.47%)	0.54 LCL: 0.25 UCL: 1.14 *p* = 0.11	M: 3 F: 4	1.07 LCL: 0.24 UCL: 4.82 *p* = 1.00	N/S	N/S	N/A	0 (0)
Pooled population	All modern populations included in statistical analysis	11,006 M: 4347 ^‡^ F: 4886 ^‡^	298 (2.71%)	N/A	M: 82 ^‡^ F: 180 ^‡^	0.50 ^‡^ LCL: 0.39 ^‡^ UCL: 0.65 ^‡^ *p* = 0.00 ^‡^	R: 2482 ^‡^ L: 2481^‡^	R: 77 ^‡^ L: 54 ^‡^	1.44 ^‡^ LCL: 1.01 ^‡^ UCL: 2.05 ^‡^ *p* = 0.05 ^‡^	1
First author, publication year, identifier	Qualification type (population/cases of birooted canines)	Number of mandibular canines (in M/F)	Birooted canines	Odds ratio per population (95% CI, *p*)	Birooted canines in M/F	M/F odds ratio (95% CI, *p*)	Number of R/L canines	Birooted R/L canines	R/L odds ratio (95% CI, *p*)	Three-rooted (and more-rooted) canines

Labels: CI—confidence interval; F—female; M—male; N/A—not applicable; N/S—not specified; L—left; LCL—lower confidence interval; R—right; U—unknown sex; UCL—upper confidence interval. Footnotes: *—estimated values; †—inferred from context; ‡—excluding studies with no available data.

## Data Availability

All collected data are included in the content of this article and Supplementary Materials. PROSPERO registration number (ID): CRD420251138164.

## Supplementary Materials

The following supporting information can be downloaded at: https://www.mdpi.com/article/10.3390/jcm15093381/s1.

## Appendix A

**Table A1:** Studies excluded at full-text assessment stage.

First Author, Publication Year	Digital Object Identifier (DOI) or Alternative	Reason for Exclusion
Antonialli, 2021	10.51929/jms.38.34.2021	No quantitative assessment of the root number
De Koninck, 2021	10.24873/j.rpemd.2021.12.852	No quantitative assessment of the root number
Manhaes-Caldas, 2025	10.1590/0103-644020256063	No quantitative assessment of the root number
Cruz-Cueva, 2022	10.35381/s.v.v6i3.2316	A review
Oporto, 2010	10.4067/S0717-95022010000300046	Only 3 cases described
Pevalek, 1995	N/A	No quantitative assessment of the root number
Ramirez-Sotelo, 2013	N/A	Only 1 case described
Ahmed, 2016	10.1111/iej.12508	A review
Çelik, 2024	10.61139/ijdor.1581558	Only 8 cases described, with no birooted canines recorded
Peiris, 2008	10.1537/ase.070723	No mandibular canines assessed
Sert, 2004	10.1097/00004770-200406000-00004	No quantitative assessment of the root number and no birooted canine recorded
Silva, 2012	10.21726/rsbo.v9i4.1030	Only 1 case described
Onda, 1985	N/A	A review
Zivanovic, 2017	10.13140/RG.2.2.26607.94886	A poster
Kuštra, 2022	N/A	A master’s thesis
Karobari, 2020	N/A	A master’s thesis
Lamanna, 2018	N/A	No quantitative assessment of the tooth number
Aminsobhani, 2013	PMID: 24396355	Quantitative data according to birooted canines not sufficient enough for analysis
Lee, 2011	10.1002/ajpa.21585	Quantitative data according to birooted canines not sufficient enough for analysis

**Table A2:** Risk of bias in reporting prevalence by country, cross-sectional studies.

First Author, Publication Year	Were the Criteria for Inclusion in the Sample Clearly Defined?	Were the Study Subjects and the Setting Described in Detail?	Was the Exposure Measured in a Valid and Reliable Way?	Were Objective, Standard Criteria Used for Measurement of the Condition?	Were Confounding Factors Identified?	Were Strategies to Deal with Confounding Factors Stated?	Were the Outcomes Measured in a Valid and Reliable Way?	Was Appropriate Statistical Analysis Used?	Overall Appraisal
Piskórz, 2023 [42]	Yes	Yes	Yes	Yes	Yes	Yes	Yes	Unclear	Low
Aydin, 2022 [43]	Yes	Yes	Yes	Yes	Yes	Yes	Yes	Yes	Low
Almohaimede, 2021 [44]	Yes	Yes	Yes	Yes	Yes	Yes	Yes	Yes	Low
Versini, 2011 [46]	Yes	Yes	Yes	Yes	Yes	Yes	Yes	Yes	Low
Doumani, 2020 [47]	Yes	Yes	Yes	Yes	Yes	Yes	Yes	Yes	Low
Khan, 2021 [48]	Yes	Yes	Yes	Yes	Yes	Yes	Yes	Yes	Low
Mashyakhy, 2019 [49]	Yes	Yes	Yes	Yes	Yes	Yes	Yes	Yes	Low
Monsarrat, 2016 [50]	Yes	Yes	Yes	Yes	Yes	Yes	Yes	Yes	Low
Mustafa, 2025 [51]	Yes	Yes	Yes	Yes	Yes	Yes	Yes	Yes	Low
Okumuş, 2022 [52]	Yes	Yes	Yes	Yes	Yes	Yes	Yes	Yes	Low
Shrestha, 2024 [53]	Yes	Yes	Yes	Yes	Yes	Yes	Yes	Yes	Low
Siddique, 2024 [54]	Yes	Yes	Yes	Yes	Yes	Yes	Yes	Yes	Low
Taha, 2024 [55]	Yes	Yes	Yes	Yes	Yes	Yes	Yes	Yes	Low
Wolf, 2021 [56]	Yes	Yes	Yes	Yes	Unclear	Unclear	Yes	Yes	Low
Shemesh, 2016 [57]	Yes	Yes	Yes	Yes	Yes	Yes	Yes	Yes	Low
Al-Dahman, 2019 [58]	Yes	Yes	Yes	Yes	Yes	Yes	Yes	Yes	Low
Alhumaidi, 2024 [59]	Yes	Yes	Yes	Yes	Yes	Yes	Yes	Yes	Low

**Table A3:** Risk of bias in reporting prevalence by country, case series.

First Author, Publication Year	Were There Clear Criteria for Inclusion in the Case Series?	Was the Condition Measured in a Standard, Reliable Way for All Participants Included in the Case Series?	Were Valid Methods Used for Identification of the Condition for All Participants Included in the Case Series?	Did the Case Series Have Consecutive Inclusion of Participants?	Did the Case Series Have Complete Inclusion of Participants?	Was There Clear Reporting of the Demographics of the Participants in the Study?	Was There Clear Reporting of Clinical Information of the Participants?	Were the Outcomes or Follow-Up Results of Cases Clearly Reported?	Was There Clear Reporting of the Presenting Site(s)/Clinic(s) Demographic Information?	Was Statistical Analysis Appropriate?	Overall Appraisal
Beltes, 2019 [45]	Yes	Yes	Yes	Unclear	Unclear	No	Yes	N/A	Yes	Yes	Some concerns

**Table A4:** Risk of bias in reporting prevalence by sex, cross-sectional studies.

First Author, Publication Year	Were the Criteria for Inclusion in the Sample Clearly Defined?	Were the Study Subjects and the Setting Described in Detail?	Was the Exposure Measured in a Valid and Reliable Way?	Were Objective, Standard Criteria Used for Measurement of the Condition?	Were Confounding Factors Identified?	Were Strategies to Deal with Confounding Factors Stated?	Were the Outcomes Measured in a Valid and Reliable Way?	Was Appropriate Statistical Analysis Used?	Overall Appraisal
Shemesh, 2016 [57]	Yes	Yes	Yes	Yes	Yes	Yes	Yes	Yes	Low
Taha, 2024 [55]	Yes	Yes	Yes	Yes	Yes	Yes	Yes	Yes	Low
Shrestha, 2024 [53]	Yes	Yes	Yes	Yes	Yes	Yes	Yes	Yes	Low
Khan, 2021 [48]	Yes	Yes	Yes	Yes	Yes	Yes	Yes	Yes	Low
Piskórz, 2023 [42]	Yes	Yes	Yes	Yes	Yes	Yes	Yes	Unclear	Low
Almohaimede, 2021 [44]	Yes	Yes	Yes	Yes	Yes	Yes	Yes	Yes	Low
Mustafa, 2025 [51]	Yes	Yes	Yes	Yes	Yes	Yes	Yes	Yes	Low
Mashyakhy, 2019 [49]	Yes	Yes	Yes	Yes	Yes	Yes	Yes	Yes	Low
Doumani, 2020 [47]	Yes	Yes	Yes	Yes	Yes	Yes	Yes	Yes	Low
Okumuş, 2022 [52]	Yes	Yes	Yes	Yes	Yes	Yes	Yes	Yes	Low
Aydin, 2022 [43]	Yes	Yes	Yes	Yes	Yes	Yes	Yes	Yes	Low
Alhumaidi, 2024 [59]	Yes	Yes	Yes	Yes	Yes	Yes	Yes	Yes	Low

**Table A5:** Risk of bias in reporting prevalence by sex, case series.

First Author, Publication Year	Were There Clear Criteria for Inclusion in the Case Series?	Was the Condition Measured in a Standard, Reliable Way for All Participants Included in the Case Series?	Were Valid Methods Used for Identification of the Condition for All Participants Included in the Case Series?	Did the Case Series Have Consecutive Inclusion of Participants?	Did the Case Series Have Complete Inclusion of Participants?	Was There Clear Reporting of the Demographics of the Participants in the Study?	Was There Clear Reporting of Clinical Information of the Participants?	Were the Outcomes or Follow-Up Results of Cases Clearly Reported?	Was There Clear Reporting of the Presenting Site(s)/Clinic(s) Demographic Information?	Was Statistical Analysis Appropriate?	Overall Appraisal
Beltes, 2019 [55]	Yes	Yes	Yes	Unclear	Unclear	No	Yes	N/A	Yes	Yes	Some concerns

**Table A6:** Risk of bias in reporting prevalence by side, cross-sectional studies.

First Author, Publication Year	Were the Criteria for Inclusion in the Sample Clearly Defined?	Were the Study Subjects and the Setting Described in Detail?	Was the Exposure Measured in a Valid and Reliable Way?	Were Objective, Standard Criteria Used for Measurement of the Condition?	Were Confounding Factors Identified?	Were Strategies to Deal with Confounding Factors Stated?	Were the Outcomes Measured in a Valid and Reliable Way?	Was Appropriate Statistical Analysis Used?	Overall Appraisal
Monsarrat, 2016 [50]	Yes	Yes	Yes	Yes	Yes	Yes	Yes	Yes	Low
Shemesh, 2016 [57]	Yes	Yes	Yes	Yes	Yes	Yes	Yes	Yes	Low
Shrestha, 2024 [53]	Yes	Yes	Yes	Yes	Yes	Yes	Yes	Yes	Low
Aydin, 2022 [43]	Yes	Yes	Yes	Yes	Yes	Yes	Yes	Yes	Low

**Table A7:** GRADE assessment.

Outcome	Number of Studies	Risk of Bias	Inconsistency	Indirectness	Imprecision	Publication Bias	Certainty of the Evidence *
Prevalence of birooted canines	19 observational studies	Low	Serious	Not serious	Not serious	Not serious	Very low
Sexual dimorphism	12 observational studies	Low	Serious	Not serious	Not serious	Not serious	Very low
Bilateral symmetry	4 observational studies	Low	Serious	Not serious	Serious	Serious	Very low

* All included studies are observational; therefore, the baseline quality of evidence is “low quality”.

## References

[1] Nino-Barrera J., Alzate-Mendoza D., Olaya-Abril C., Gamboa-Martinez L.F., Guamán-Laverde M., Lagos-Rosero N., Romero-Diaz A.C., Duran N., Vanegas-Hoyose L. (2022). Atypical Radicular Anatomy in Permanent Human Teeth: A Systematic Review. Crit. Rev. Biomed. Eng..

[2] Laganà G., Venza N., Borzabadi-Farahani A., Fabi F., Danesi C., Cozza P. (2017). Dental Anomalies: Prevalence and Associations between Them in a Large Sample of Non-Orthodontic Subjects, a Cross-Sectional Study. BMC Oral Health.

[3] Khanna A.B. (2020). Applications of Cone Beam Computed Tomography in Endodontics. Evid.-Based Endod..

[4] Svetlozarova S. (2019). Cone Beam Computed Tomography as a Diagnostic Tool in Endodontics—A Review of the Literature. Adv. Dent. Oral Health.

[5] Flores-Mir C., Rosenblatt M.R., Major P.W., Carey J.P., Heo G. (2014). Measurement Accuracy and Reliability of Tooth Length on Conventional and CBCT Reconstructed Panoramic Radiographs. Dent. Press. J. Orthod..

[6] Turosz N., Chęcińska K., Chęciński M., Sielski M., Sikora M. (2024). Evaluation of Dental Panoramic Radiographs by Artificial Intelligence Compared to Human Reference: A Diagnostic Accuracy Study. J. Clin. Med..

[7] Martins L.A.C., Brasil D.M., Forner L.A., Viccari C., Haiter-Neto F., Freitas D.Q., Oliveira M.L. (2021). Does Dose Optimisation in Digital Panoramic Radiography Affect Diagnostic Performance?. Clin. Oral Investig..

[8] Charuakkra A., Mahasantipiya P., Lehtinen A., Koivisto J., Järnstedt J. (2023). Comparison of Subjective Image Analysis and Effective Dose between Low-Dose Cone-Beam Computed Tomography Machines. Dentomaxillofac. Radiol..

[9] Iosif L., Dimitriu B., Niţoi D.F., Amza O. (2023). Endodontic Dentistry: Analysis of Dentinal Stress and Strain Development during Shaping of Curved Root Canals. Healthcare.

[10] Xu T., Gao X., Fan W., Fan B. (2020). Micro-Computed Tomography Evaluation of the Prevalence and Morphological Features of Apical Bifurcations. J. Dent. Sci..

[11] Mamat R., Nik Abdul Ghani N.R. (2023). The Complexity of the Root Canal Anatomy and Its Influence on Root Canal Debridement in the Apical Region: A Review. Cureus.

[12] Sakkir N., Thaha K., Nair M., Joseph S., Christalin R. (2014). Management of Dilacerated and S-Shaped Root Canals—An Endodontist’s Challenge. J. Clin. Diagn. Res..

[13] Shantiaee Y., Zandi B., Hosseini M., Davoudi P., Farajollahi M. (2024). Quality of Root Canal Filling in Curved Canals Utilizing Warm Vertical Compaction and Two Different Single Cone Techniques: A Three-Dimensional Micro-Computed Tomography Study. J. Dent..

[14] Mathew J., Devadathan A., Syriac G., Shamini S. (2015). Root Canal Treatment of a Maxillary First Premolar with Three Roots. J. Pharm. Bioallied Sci..

[15] Huang D., Wang X., Liang J., Ling J., Bian Z., Yu Q., Hou B., Chen X., Li J., Ye L. (2024). Expert Consensus on Difficulty Assessment of Endodontic Therapy. Int. J. Oral Sci..

[16] Arora A., Acharya S.R., Sharma P. (2015). Endodontic Treatment of a Mandibular First Molar with 8 Canals: A Case Report. Restor. Dent. Endod..

[17] Darwish G. (2024). Radiographic Evaluation Factors That Influence the Decision of the Tooth Extraction Method. Cureus.

[18] Lima-Sánchez B., Hermida-Cabrera P., Montoya-Salazar V., Oliveros-López L.-G., Alomar-Velasco P., Serrera-Figallo M.-A., Torres-Lagares D., Baus-Domínguez M. (2024). Estimating the Extraction Time of an Upper Third Molar: Proposal and Validation of Results. Diagnostics.

[19] Lindahl O., Ventä I. (2023). Level of Difficulty of Tooth Extractions among Roughly 100,000 Procedures in Primary Care. Clin. Oral Investig..

[20] Bell G.W., Rodgers J.M., Grime R.J., Edwards K.L., Hahn M.R., Dorman M.L., Keen W.D., Stewart D.J.C., Hampton N. (2003). The Accuracy of Dental Panoramic Tomographs in Determining the Root Morphology of Mandibular Third Molar Teeth before Surgery. Oral Surg. Oral Med. Oral Pathol. Oral Radiol. Endodontol..

[21] Cicciù M., Bramanti E., Signorino F., Cicciù A., Sortino F. (2013). Experimental Study on Strength Evaluation Applied for Teeth Extraction: An In Vivo Study. Open Dent. J..

[22] Gazal G., Omar E., Fareed W., Alsharif A., Bahabri R. (2020). Impact of Maxillary Teeth Morphology on the Failure Rate of Local Anesthesia. Saudi J. Anaesth..

[23] Lin C.-Y., Yu Y.-Y. (2025). Mandibular Left First Premolar with Three Roots and Three Canals: A Case Report. World J. Clin. Cases.

[24] Mohamed A.G., Ibrahim A.E.E., Abdusalam A., Milad M. (2023). Tooth Extraction Using Vertical, Conventional, and Surgical Techniques in Sebha Dental College: A Descriptive “Cross-Sectional Study”. Open Dent. J..

[25] Tatli U., Cavana A., Tukel H.C., Benlidayi M.E. (2025). Effects of Bone Augmentation on Implant Success and Survival: A Retrospective Analysis With 6-Year Mean Follow-Up. Clin. Implant. Dent. Rel Res..

[26] Dam V.V., Trinh H.A., Rokaya D., Trinh D.H. (2022). Bone Augmentation for Implant Placement: Recent Advances. Int. J. Dent..

[27] Jensen A.T., Jensen S.S., Worsaae N. (2016). Complications Related to Bone Augmentation Procedures of Localized Defects in the Alveolar Ridge. A Retrospective Clinical Study. Oral Maxillofac. Surg..

[28] Akören A.C., Karaačaçliočlu L. (1995). Comparison of the Electromyographic Activity of Individuals with Canine Guidance and Group Function Occlusion. J. Oral Rehabil..

[29] Aldowish A.F., Alsubaie M.N., Alabdulrazzaq S.S., Alsaykhan D.B., Alamri A.K., Alhatem L.M., Algoufi J.F., Alayed S.S., Aljadani S.S., Alashjai A.M. (2024). Occlusion and Its Role in the Long-Term Success of Dental Restorations: A Literature Review. Cureus.

[30] Oztol E., Uctasli S. (2024). Mandibular Canine Root-Supported Overdentures: A Case Report. Int. Dent. J..

[31] Limpuangthip N., Somkotra T., Arksornnukit M. (2019). Impacts of Denture Retention and Stability on Oral Health-Related Quality of Life, General Health, and Happiness in Elderly Thais. Curr. Gerontol. Geriatr. Res..

[32] Bouanane B., Assraoui K.E., Oubbaih A., Kaoun K., Bellemkhannate S. (2024). Treatment Approaches to Improve Retention and Stability of Mandibular Complete Dentures. Open Access Libr. J..

[33] Jacobson T.E., Krol A.J. (1983). A Contemporary Review of the Factors Involved in Complete Denture Retention, Stability, and Support. Part I: Retention. J. Prosthet. Dent..

[34] Tiwari B., Ladha K., Lalit A., Dwarakananda Naik B. (2014). Occlusal Concepts in Full Mouth Rehabilitation: An Overview. J. Indian Prosthodont. Soc..

[35] Sanz-Sánchez I., Sanz-Martín I., Ortiz-Vigón A., Molina A., Sanz M. (2022). Complications in Bone-grafting Procedures: Classification and Management. Periodontol. 2000.

[36] Page M.J., McKenzie J.E., Bossuyt P.M., Boutron I., Hoffmann T.C., Mulrow C.D., Shamseer L., Tetzlaff J.M., Akl E.A., Brennan S.E. (2021). The PRISMA 2020 Statement: An Updated Guideline for Reporting Systematic Reviews. BMJ.

[37] Page M.J., Moher D., Bossuyt P.M., Boutron I., Hoffmann T.C., Mulrow C.D., Shamseer L., Tetzlaff J.M., Akl E.A., Brennan S.E. (2021). PRISMA 2020 Explanation and Elaboration: Updated Guidance and Exemplars for Reporting Systematic Reviews. BMJ.

[38] Ouzzani M., Hammady H., Fedorowicz Z., Elmagarmid A. (2016). Rayyan—A Web and Mobile App for Systematic Reviews. Syst. Rev..

[39] JBI Critical Appraisal Tools|JBI. https://jbi.global/critical-appraisal-tools.

[40] Aragon T.J., Fay M.P., Wollschlaeger D., Omidpanah A. (2020). R Package.

[41] Haddaway N.R., Page M.J., Pritchard C.C., McGuinness L.A. (2022). PRISMA2020: An R Package and Shiny App for Producing PRISMA 2020-compliant Flow Diagrams, with Interactivity for Optimised Digital Transparency and Open Synthesis. Campbell Syst. Rev..

[42] Piskórz M., Futyma-Gąbka K., Różyło-Kalinowska I. (2023). Prevalence of Two-Rooted and One-Rooted Mandibular Canines with Two Root Canals in Poland, Assessed Using CBCT: A Preliminary Study. Dent. Med. Probl..

[43] Aydın H. (2023). Comparing the Crown and Root Metric Properties of Double-Rooted and Single-Rooted Mandibular Canine Teeth. Oral Radiol..

[44] Almohaimede A.A., Alqahtani A.A., Alhatlani N.M., Alsaloom N.S., Alqahtani S.A. (2021). Interpretation of Root Canal Anatomy of Maxillary and Mandibular Permanent Canines in Saudi Subpopulation: A Cone-Beam Computed Tomography (CBCT) Study. Int. J. Dent..

[45] Beltes P., Kantilieraki E., Kalaitzoglou M., Beltes C., Angelopoulos C. (2019). Mandibular Canines with Additional Roots: An *Ex Vivo* Study of the External and Internal Morphology. Aust. Endod. J..

[46] Versiani M.A., Pécora J.D., Sousa-Neto M.D. (2011). The Anatomy of Two-Rooted Mandibular Canines Determined Using Micro-Computed Tomography. Int. Endod. J..

[47] Doumani M., Habib A., Alhalak A., Al-Nahlawi T., Al Hussain F., Alanazi S. (2020). Root Canal Morphology of Mandibular Canines in the Syrian Population: A CBCT Assessment. J. Fam. Med. Prim. Care.

[48] Khan N.B., Azhar M., Abbasi N., Mehmood B. (2021). Frequency of Two Roots in Permanent Mandibular Canine of Pakistani Population: A Cone Beam Computerized Tomography (CBCT) Study. J. Univ. Med. Dent. Coll..

[49] Mashyakhy M. (2019). Prevalence of a Second Root and Canal in Mandibular and Maxillary Canines in a Saudi Arabian Population: A Cone-Beam Computed Tomography Study. J. Contemp. Dent. Pract..

[50] Monsarrat P., Arcaute B., Peters O.A., Maury E., Telmon N., Georgelin-Gurgel M., Maret D. (2016). Interrelationships in the Variability of Root Canal Anatomy among the Permanent Teeth: A Full-Mouth Approach by Cone-Beam CT. PLoS ONE.

[51] Mustafa M., Karobari M.I., Al-Maqtari A.A.A., Abdulwahed A., Almokhatieb A.A., Almufleh L.S., Hashem Q., Alsakaker A., Alam M.K., Ahmed H.M.A. (2025). Investigating Root and Canal Morphology of Anterior and Premolar Teeth Using CBCT with a Novel Coding Classification System in Saudi Subpopulation. Sci. Rep..

[52] Okumuş Ö., Çoban Kanyılmaz A.N. (2022). Assessment of Root Canal Anatomy of Maxillary and Mandibular Canine Teeth: A Cone-Beam Computed Tomography Study. Odovtos-Int. J. Dent. Sci..

[53] Shrestha K., Shubham S., Ahmed S., Gautam V. (2024). Variations in the Root Form and Root Canal Morphology of Permanent Mandibular Canine. J. Nepal. Health Res. Counc..

[54] Siddique S.N., Babar P., Ghazanfar Z., Kayani J.A. (2024). Root Canal Morphology of Permanent Mandibular Anterior Teeth in a Pakistani Population: A Cone Beam Computed Tomography Assessment. Pak. J. Med. Sci..

[55] Taha N.A., Makahleh N., Hatipoglu F.P. (2024). Root Canal Morphology of Anterior Permanent Teeth in Jordanian Population Using Two Classification Systems: A Cone-Beam Computed Tomography Study. BMC Oral Health.

[56] Wolf T.G., Anderegg A.L., Haberthür D., Khoma O.-Z., Schumann S., Boemke N., Wierichs R.J., Hlushchuk R. (2021). Internal Morphology of 101 Mandibular Canines of a Swiss-German Population by Means of Micro-CT: An Ex Vivo Study. Sci. Rep..

[57] Shemesh A., Levin A., Katzenell V., Itzhak J.B., Avraham Z., Levinson O., Solomonov M. (2016). Root anatomy and root canal morphology of mandibular canines in Israeli population. Refuat Hapeh Vehashinayim (1993).

[58] Al-Dahman Y., Alqedairi A., Alfawaz H., Alnassar F., Al-Jebaly A. (2019). Cone-Beam Computed Tomographic Evaluation of Root Canal Morphology of Mandibular Canines in a Saudi Subpopulation. Saudi Endod. J..

[59] Alhumaidi A.M., Mirza M.B., Karobari M.I., Abuelqomsan M.A., Hashem Q., Aldaijy M.T., Albarr N.Y., Aldaijy R.T., Al Moaleem M. (2025). Classifying the Internal Anatomy of Anterior Teeth in the Yemeni Population Using Two Systems: A Retrospective CBCT Study. Odontology.

[60] Martins J.N.R., Ensinas P., Chan F., Babayeva N., Von Zuben M., Berti L., Lam E.W.N., Antúnez M., Pei F., Mendez De La Espriella C. (2024). Worldwide Anatomic Characteristics of the Mandibular Canine—A Multicenter Cross-Sectional Study with Meta-Analysis. J. Endod..

[61] Lee C., Scott G.R. (2011). Brief communication: Two-rooted lower Canines—A European trait and sensitive indicator of admixture across Eurasia. Am. J. Phys. Anthropol..

